# Dengue Encephalitis Versus Japanese Encephalitis in Cases of Dengue Fever With Altered Sensorium: A Diagnostic Dilemma

**DOI:** 10.7759/cureus.50146

**Published:** 2023-12-07

**Authors:** Ambuj Yadav, Vikas Chandra Vidyarthi, Deepak Bhagchandani, Mahak Lamba, Namrta Yadav

**Affiliations:** 1 Internal Medicine, King George's Medical University, Lucknow, IND; 2 Medicine, King George's Medical University, Lucknow, IND; 3 Gastroenterology and Hepatology, King George's Medical University, Lucknow, IND; 4 Gynecology, Autonomous State Medical College, Hardoi, IND

**Keywords:** igm dengue, ns1 antigen, cross-reactivity, coinfection, dengue encephalitis, japanese encephalitis, dengue fever

## Abstract

Dengue and Japanese encephalitis (JE) are diseases that often conquer the top headlines in the leading newspapers during epidemics. Although recovery is the rule in most dengue cases, some unfortunately land up with multiple organ dysfunction syndromes, get critical, and even succumb to death. The main risk here is bleeding due to thrombocytopenia and platelet dysfunction. On the other hand, JE often presents with acute encephalitis syndrome (AES). We report a confirmed case of dengue (NS1 reactive, IgM dengue positive) by enzyme-linked immunosorbent assay (ELISA) who developed sudden onset altered sensorium. Non-contrast computed tomography (NCCT) head was done, which showed an infarct in the right gangliocapsular region with normal-sized ventricles. The patient had deteriorated in the past four days, which warranted a repeat NCCT head, revealing dilated ventricles and hemorrhagic transformation in the old infarct with surrounding edema. CSF viral markers were suggestive of IgM anti-JE virus positive. An MRI brain was planned but could not be done due to the deteriorating condition of the patient. Unfortunately, the patient landed up with multiple organ dysfunction syndrome and succumbed to death.

## Introduction

Rain has always been a symbol of joy that often creates a smiling curve on the face of *Homo sapiens*. Joy is followed by sorrow and vice versa; probably, this is the way nature maintains the equilibrium between the two. The post-rainy season creates a favorable biome for the microorganisms to grow and transmit disease, creating havoc in mankind and thus marking the beginning of sorrow in the lives of humans.

Dengue and Japanese encephalitis (JE) have been endemic in almost all corners of India, and the reports of their outbreaks in the news seem to be nothing more than cliche. Globally, JE is responsible for approximately 68,000 clinical cases every year [1]. Dengue virus (DV) causes classical dengue fever, dengue hemorrhagic fever, and dengue shock syndrome, while JE virus manifests encephalitis. Although the DV may cause encephalitis mimicking JE, clinical features, seasonality, and geographical locations for DV and JE virus infections often coincide [2].

Both dengue and JE transmission intensify during the rainy season and are spread by the bite of infected mosquitoes. Both share antigenic epitopes that might cross-react with others in serological tests, referred to as cross-reacting antibodies. In regions endemic to DV and JE, accurate diagnosis by serological methods is often challenging, as is often complicated by cross-reactivity [3].

## Case presentation

A 23-year-old male resident of Sultanpur, Uttar Pradesh, was referred from a private hospital to our emergency room. He presented with chief complaints of a fever of one month associated with chills and rigor, weakness in the left side of the body, and altered sensorium for four days, followed by gradual progressive breathlessness. The patient was being treated by a local practitioner in a private hospital for a dengue viral infection (NS1 reactive by enzyme-linked immunosorbent assay (ELISA)) for 10 days. The patient deteriorated in the last four days when the NCCT (non-contrast computed tomography) head was done, and the patient was referred to the tertiary care center for further management (Table 1).

**Table 1:** Vitals at presentation of the patient. GCS: Glasgow Coma Scale.

GCS	E3V2M2
Blood pressure	132/68 mmHg
SpO_2_	96% on 15 L Hudson mask
Pulse rate	158/min
Chest	Bilateral coarse crepts
Pupil	Left-sided: dilated and non-reacting to light Right-sided: normal size normal reaction to light
Power	Upper limb (right): 5/5; upper limb (left): 2/5; lower limb (right): 5/5; lower limb (left): 2/5
Plantar	Withdrawal

**Figure 1:**
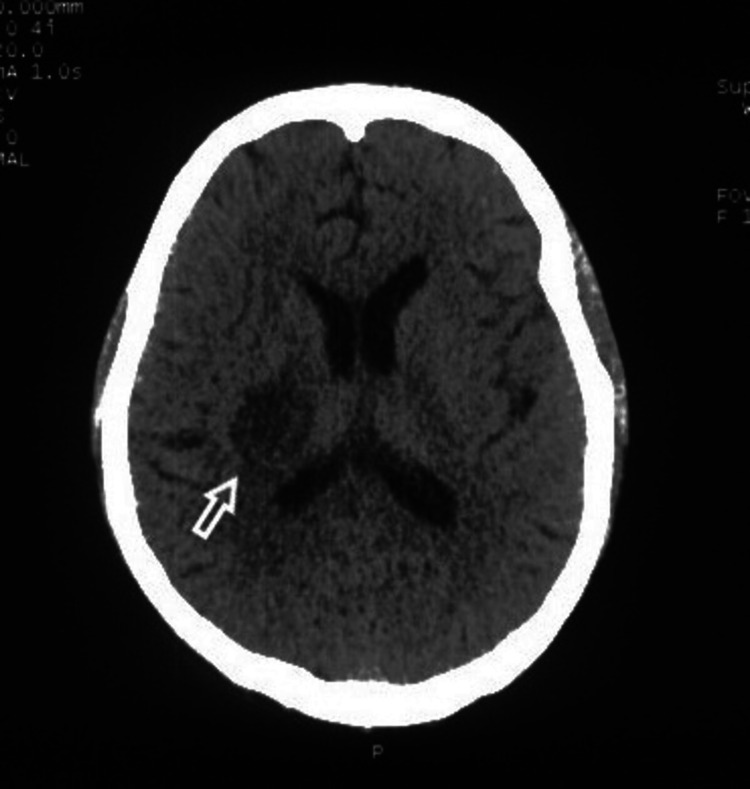
Arrow showing right gangliocapsular infarct on NCCT head at presentation. NCCT: non-contrast computed tomography.

NCCT head of the patient revealed a right-sided gangliocapsular infarct, which was done one week before admission to our tertiary care center (Figure 1). 

**Figure 2:**
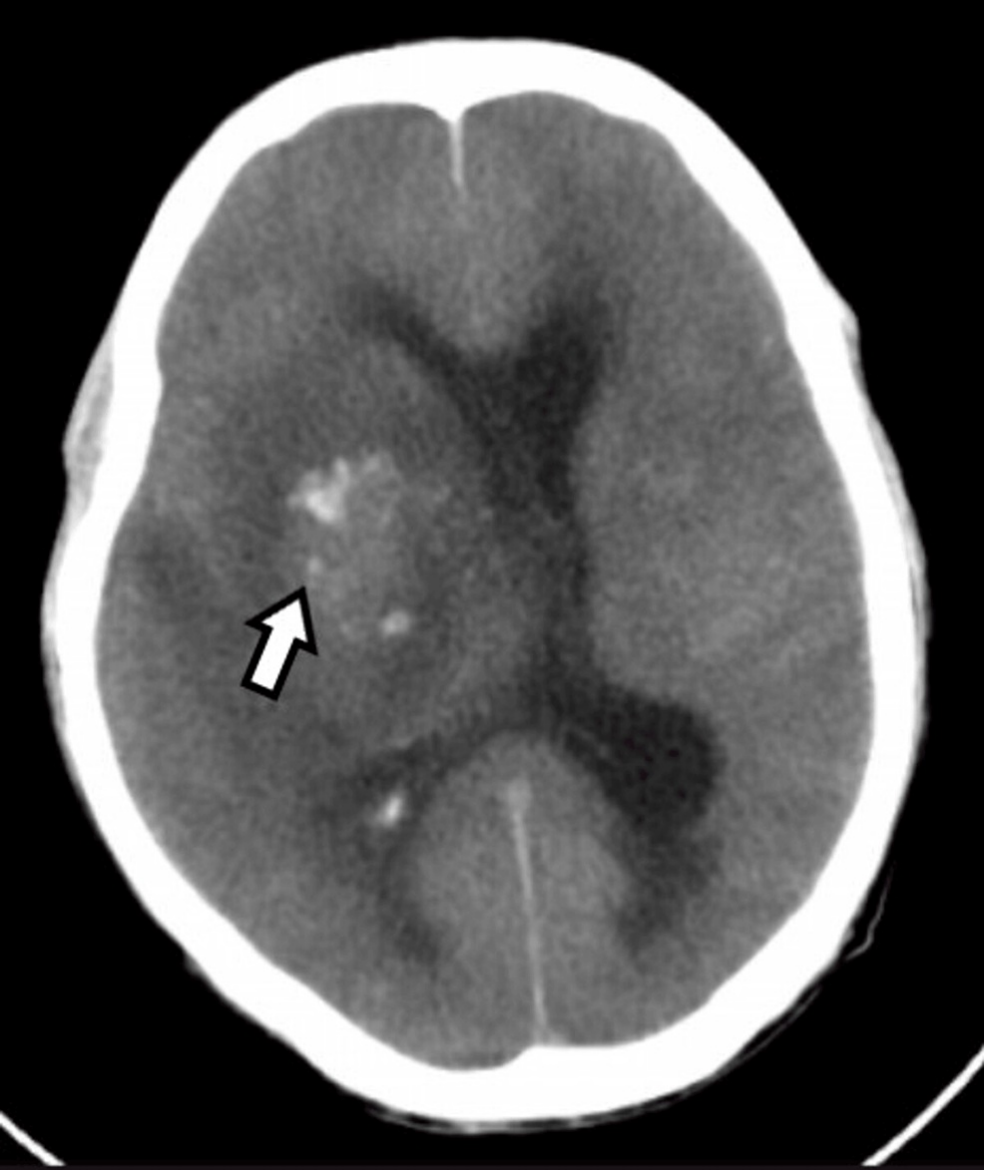
Arrow showing hemorrhagic transformation with dilated ventricles and mid line shift.

The patient was intubated due to a poor Glasgow Coma Scale (GCS) and respiratory failure, placed on ventilatory support, and shifted to the ICU. Routine investigations were conducted along with a test for serum IgM dengue, which came out to be positive by ELISA (NC: 0.066, OD: 0.899). A chest X-ray was performed, suggesting right-sided heterogeneous opacity attributed to pneumonia, probably aspirational due to the low GCS of the patient. CSF analysis was performed due to pyrexia with altered sensorium, which revealed 80 cells (lymphocytic predominance), protein 319.5 mg%, and glucose 44.7 mg%. CSF viral markers were tested, which revealed IgM JE virus positive (NC: 0.096, OD: 0.923). The patient's GCS score did not improve, and a repeat NCCT head was done, which revealed a hemorrhagic transformation in the previous infarct attributed to platelet dysfunction and thrombocytopenia due to dengue arboviral infection (Figure 2).

The brain MRI was scheduled to determine the affected anatomical locations in the brain, which could probably project the culprit microorganisms responsible for creating this illness in this young lad and may give us a clue to this diagnostic dilemma. The plan of MRI brain could not be accomplished due to the labile general condition of the patient. The patient was managed conservatively by IV fluids and antibiotics, but the GCS of the patient did not improve, and the patient deteriorated, landed up in multiple organ dysfunction syndrome, and unfortunately succumbed to death.

## Discussion

In India and other parts of Southeast Asia, DV infection and JE very frequently co-exist in the same population. Coinfection of both the DV and JE viruses has been very commonly found in the paddy-growing Terai regions of Uttar Pradesh. It is estimated that there are 390 million dengue infections annually, and the number of dengue cases has increased exponentially worldwide [4]. The mortality of JE is estimated to be about 20-30% [5].

DV and JE often share a common clinical scenario that complicates the clinical diagnosis, making the lab diagnosis a confirmatory entity to discriminate between the two culprit viruses. A prior infection with another flavivirus is protective in JE.

Several studies show that the two viruses can be differentiated by comparing the adjusted optical absorbance of anti-DV IgM with that of anti-JEV IgM. The virus for which titers are high is considered to be the infecting virus [6]. Plaque reduction and neutralization test (PRNT) is the most specific assay for the determination of DV and JE-neutralizing antibodies and remains the ultimate tool for distinguishing between the two viruses [7].

## Conclusions

Fever is often overlooked in rural areas, which delays the arrival of the patient to the hospital and ultimately increases the morbidity and mortality of the patients contributing to the case fatality rates. Awareness programs at the district level, block level, and even the village level might aid in overcoming this cumbersome havoc. Possibilities of cross-reaction, sequential infection, and co-infection should be kept in areas where both the notorious viruses exist in the same microbiome. Though the financial and health setup constraints often predominate, on the face of the map of India, in a tertiary care center it is the wise clinical decision of the clinician that cracks such diagnostic dilemma.
